# Publisher Correction: Orexin signaling modulates synchronized excitation in the sublaterodorsal tegmental nucleus to stabilize REM sleep

**DOI:** 10.1038/s41467-020-18722-z

**Published:** 2020-09-25

**Authors:** Hui Feng, Si-Yi Wen, Qi-Cheng Qiao, Yu-Jie Pang, Sheng-Yun Wang, Hao-Yi Li, Jiao Cai, Kai-Xuan Zhang, Jing Chen, Zhi-An Hu, Fen-Lan Luo, Guan-Zhong Wang, Nian Yang, Jun Zhang

**Affiliations:** Department of Physiology, Third Military Medical University, 400038 Chongqing, P.R. China

**Keywords:** Hypocretin, Hypocretin, Orexin, Orexin, REM sleep

Correction to: *Nature Communications* 10.1038/s41467-020-17401-3, published online 21 July 2020.

In the original version of this Article, the captions of Figs. 6i, 7f, and 8j were inadvertently changed during the production process from ‘EEG theta power’ to ‘EMG theta power’. In addition, in Fig. 7c, the separated left captions ‘EMG’ and ‘Frequency (Hz)’ were inadvertently merged as a single term during the production process. They have now been separated more clearly to label the raw data and frequency plot separately. The Peer Review File was also formatted incorrectly. This has now been corrected in the PDF and HTML versions of the Article.

