# Supplementary material for: Orexin signaling modulates synchronized excitation in the sublaterodorsal tegmental nucleus to stabilize REM sleep
Source: Nat Commun. 2020 Jul 21;11:3661. doi: 10.1038/s41467-020-17401-3 (PMC7374574; doi:10.1038/s41467-020-17401-3)
Supplement: Supplementary file 1 — Supplemental Information [file 41467_2020_17401_MOESM1_ESM.pdf]

Supplementary Information

Orexin signaling modulates synchronized excitation in the sublaterodorsal tegmental nucleus to stabilize REM sleep

Feng et al.

Supplementary Figure 1

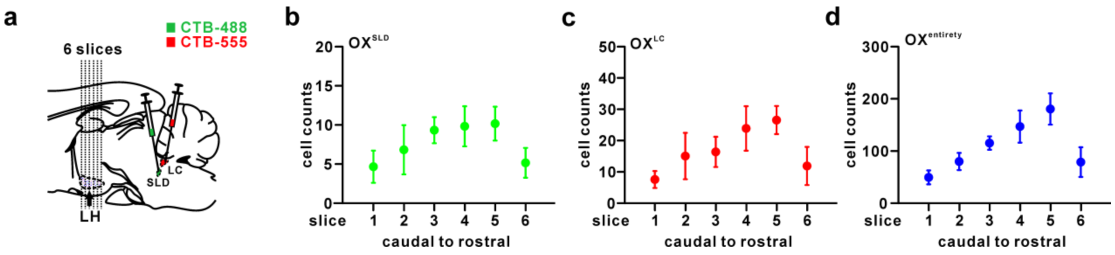

Supplementary Fig. 1 SLD-projecting orexin neurons are sporadically distributed in the hypothalamus

a Schematic for CTB retrograde tracing and cell counting strategy in rats. The number of SLD-projecting orexin neurons (OX^{SLD}), LC-projecting orexin neurons (OX^{LC}), and the orexin neuron entirety ($OX^{entirty}$), were counted in the orexin neuron containing region of the lateral hypothalamus (LH) from 6 coronal sections with a distance of 200 μm of each rat (bregma, AP: -2.30 to -3.80 mm).

b-d Distribution of the OX^{SLD} neurons **(b)**, OX^{LC} neurons **(c)**, and $OX^{entirety}$ neurons **(d)** in the caudal to rostral axis (analysis of 3902 orexin neurons from 6 rat brains). Both the OX^{SLD} and OX^{LC} neurons exhibited similar distribution patterns to the $OX^{entirety}$ neurons, suggesting that they were all sporadically distributed.

Data represent mean ± SEM. Source data are provided as a Source Data file.

Supplementary Figure 2

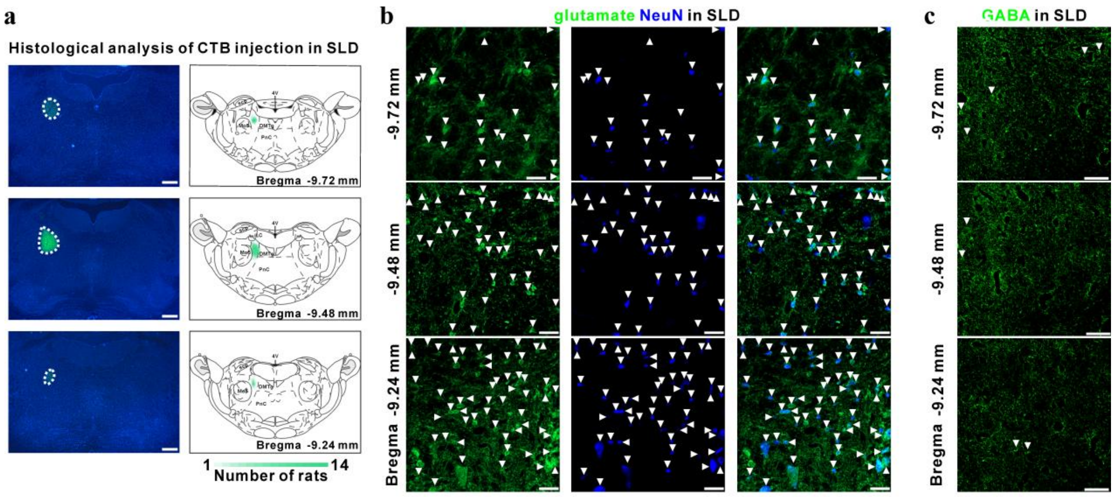

Supplementary Fig. 2 Location of the CTB injected region in the SLD and glutamate/GABA immunostaining in this region

a (Left) Example coronal images from a CTB-488 (green) injected rat showing that the CTB diffusion was within the borders (bregma, AP: -9.20 to 9.80 mm; see Methods) of the SLD region. All scale basr: 500 μm. (Right) Overlay for a series of CTB injection regions from 14 rats.

b Double immunostaining of glutamate (green) and NeuN (blue) in this SLD region showing that the majority of the SLD neurons ($NeuN^+$) were glutamatergic. Arrow heads: $NeuN^+$/$Glutamate^+$ neurons. All scale bars: 20 μm.

c Consistent with GAD-67 immunostaining, GABA (green) antibody also detected few GABAergic somas in this SLD region. Arrow heads: putative $GABA^+$ somas. All scale bars: 20 μm. It seemed that the small number of GABAergic SLD neurons had smaller soma size than glutamatergic neurons, similar to a previous report[1].

Supplementary Figure 3

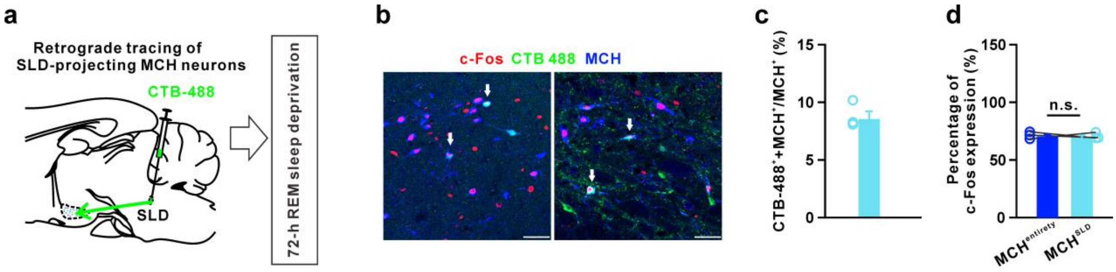

Supplementary Fig. 3 Identification of the SLD-projecting melanin-concentrating hormone (MCH) neurons and their REM sleep related activities

a CTB-488 was injected in the unilateral SLD of rats. After the rebound from 72-hour REM sleep deprivation, MCH and c-Fos immunostaining were performed to identify the SLD-projecting MCH (MCH^{SLD}) neurons, and c-Fos expression between the MCH^{SLD} neurons and the total MCH population ($MCH^{entirety}$) was compared. EEG/EMG recordings showed that the amount of REM sleep was enriched to 33.6 ± 3.2%, during the 2.5-h rebound period after the RSD (n = 3 rats).

b Representative images showing the distribution of the $MCH^{entirety}$ (MCH^+) and MCH^{SLD} (MCH^+/CTB-488^+) neurons (CTB-488, green; MCH, blue), and their c-Fos (red) expression after the REM sleep rebound. C-Fos expression were observed in both the $MCH^{entirety}$ and MCH^{SLD} neurons (arrows indicated) neurons. Scale bars, 50 μm.

c The MCH^{SLD} neurons accounted for 8.5 ± 0.7% of the total MCH population.

d C-Fos expression was not significantly different between the $MCH^{entirety}$ and

MCH^{SLD} neurons after the REM sleep rebound ($MCH^{entirety}$: 71.4 ± 1.8%, MCH^{SLD}: 70.8 ± 1.6%; n = 3 rats, two-sided pair-t test; t_2 = 0.167, P = 0.883; analysis of 5607 MCH neurons and 468 MCH^{SLD} neurons from 3 rat brains).

Data represent mean ± SEM. Source data are provided as a Source Data file.

Supplementary Figure 4

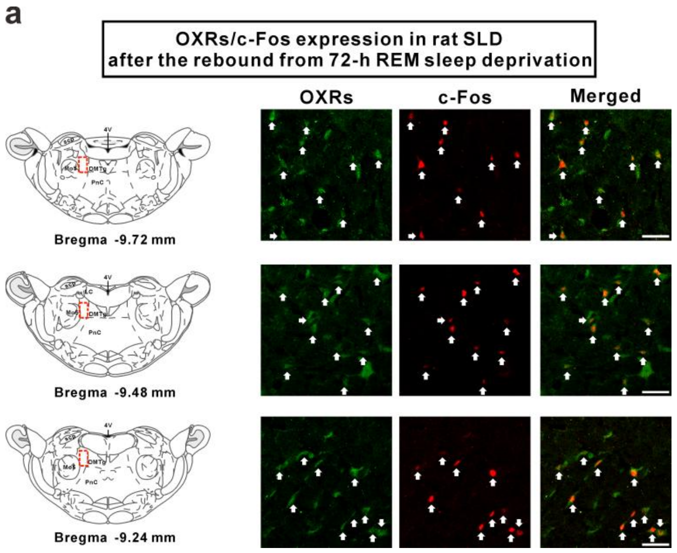

Supplementary Fig. 4 Orexin receptors are expressed in SLD REM-on neurons after the rebound period from 72-h REM sleep deprivation

a Double immunostaining of orexin receptors (OXRs, green) and c-Fos (red) were performed in the SLD region of rats after 2.5-h rebound period from 72-h REM sleep deprivation (RSD). EEG/EMG recordings showed that the amount of REM sleep was enriched to 33.6 ± 3.2%, during the 2.5-h rebound period after the RSD (n = 3 rats). The immunostaining results showed that a large majority of REM-on (c-Fos$^+$) SLD neurons also expressed OXRs. Besides, OXRs$^+$ SLD neurons without c-Fos expression were also found in this condition. All scales bars, 50 μm.

Supplementary Figure 5

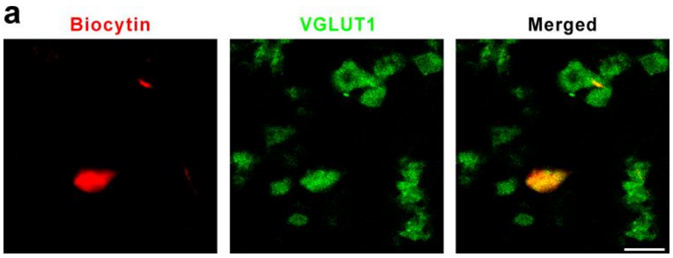

Supplementary Fig. 5 Post hoc immunostaining of VGLUT1 in patch-clamp recordings

a In 16 patch-clamp recorded rat SLD neurons with cell diameter larger than 15 μm (membrane capacitance > 80 pF), post hoc immunostaining showed that they were all pot hoc labeled by VGLUT1, suggesting that the recorded neurons in patch-clamp recordings were primarily glutamatergic neurons (See also Methods). Scale bar, 20 μm.

Supplementary Figure 6

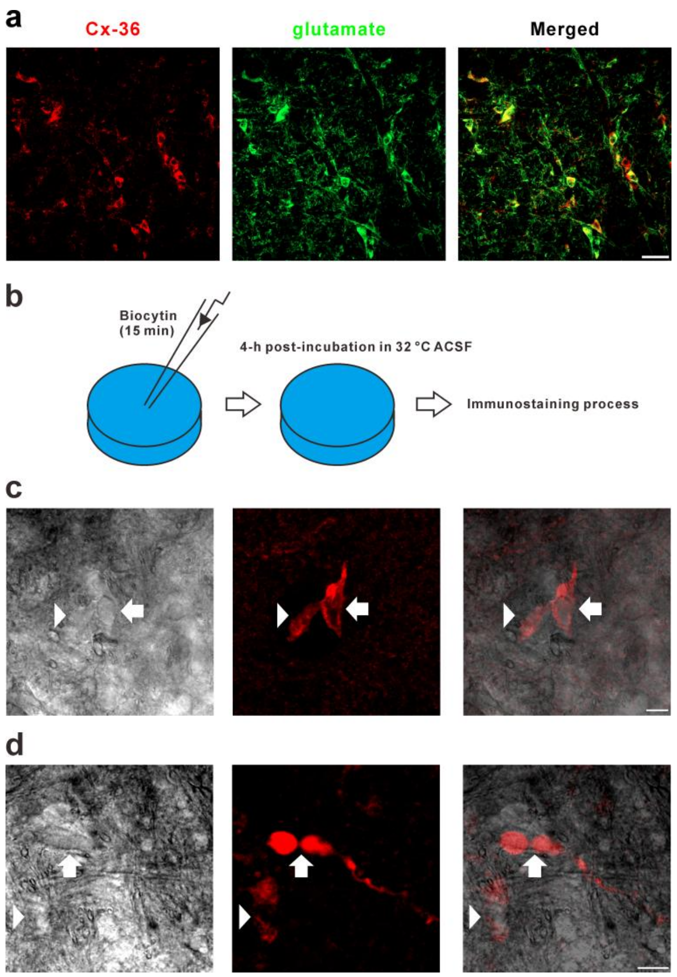

Supplementary Fig. 6 Cx-36 is expressed in the SLD glutamatergic neurons, and the spread of biocytin from the patched neuron to its neighboring neuron was observed after a 4-h post incubation

a Double immunostaining of Cx-36 (red) and glutamate (green) indicated that Cx-36 expressed at the cell membrane of the SLD glutamatergic neurons in adulthood. In addition, Cx-36 expression was also detected in the arbors of the SLD. Scale bars: 40 μm.

b Biocytin was loaded into a SLD neuron for 15 minutes. After that, the slice was post-incubated in 32 ℃ ACSF for 4 hours to facilitate the gap junction mediated spread of biocytin. Then, the slice was fixed, dehydrated and re-sectioned for the immunostaining process.

c,d After the immunostaining process, the spread of biocytin (red) from the patched neuron (arrows) to its neighboring neuron (arrow heads) was observed in the SLD in 3 of 6 tested neurons, suggesting the existence of electrical coupling in the SLD. All scale bars: 10 μm.

Supplementary Figure 7

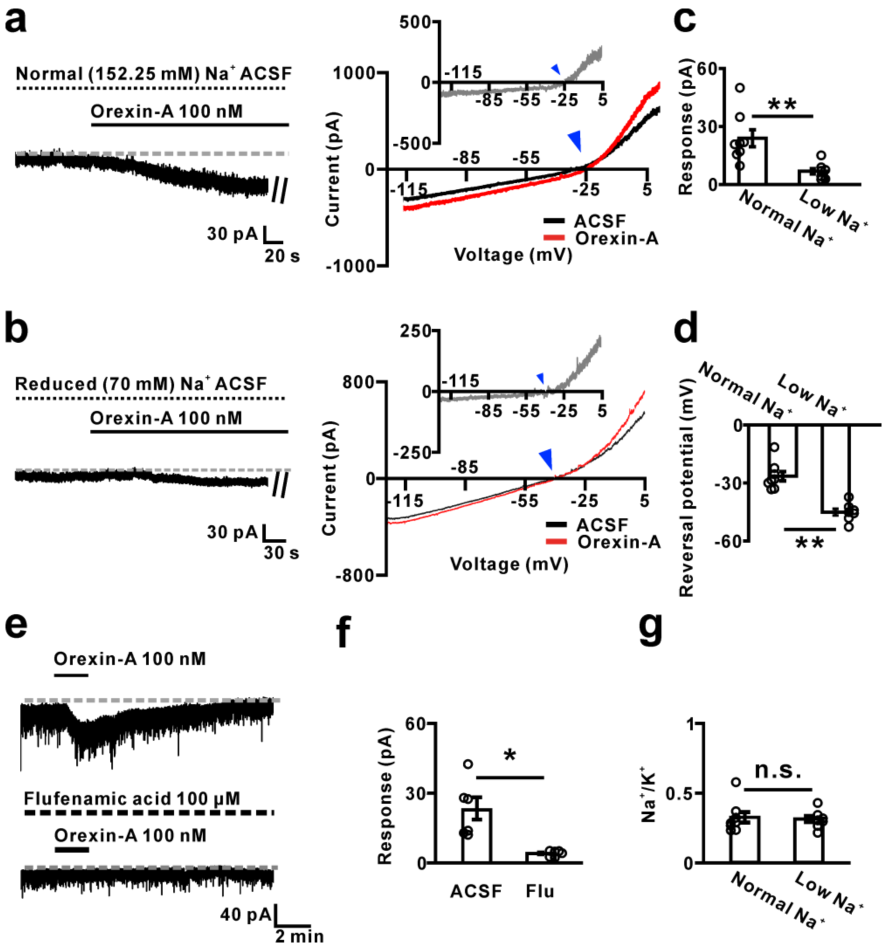

Supplementary Fig. 7 The orexin-elicited excitation of the SLD neurons is mediated by the activation of non-selective cationic conductance (NSCC)

a (Left) Representative traces showing the current elicited by orexin-A (100 nM) in normal (152.25 mM Na^+) ACSF at -70 mV in a tested SLD neuron. (Right) Current-voltage plots (I-V curves) before (black) and during (red) the application of orexin-A in this neuron revealed an equilibrium potential of -25.5 mV, near the equilibrium potential of the NSCC in normal ACSF. Insets showing the I-V relationship of the orexin-elicited net current by subtracting the control response from

the response in the presence of orexin-A.

b (Left) Representative traces showing the current elicited by orexin-A (100 nM) in reduced (70 mM) Na^+ ACSF at -70 mV in another tested SLD neuron. (Right) I-V plots before (black) and during (red) the application of orexin-A revealed an equilibrium potential of -45.7 mV in this neuron.

c Group data showing that the orexin-elicited inward current was reduced after the reduction of Na^+ concentration from 152.25 to 70 mM (152.25 mM Na^+: 23.9 ± 4.5 pA, n = 8; 70 mM Na^+: 6.9 ± 1.6 pA, n = 7; Mann-Whitney rank-sum test; z = 3.125, $P = 1.780 \times 10^{-3}$).

d Group data showing that the reversal potential of the orexin-elicited net current was shifted to a more negative value after the reduction of Na^+ concentration from 152.25 to 70 mM (152.25 mM Na^+: -26.5 ± 2.5 mV, n = 8; 70 mM Na^+: -45.1 ± 1.8 mV, n = 7; two-sided unpaired t-test; $t_{13} = 5.850$, $P = 5.684 \times 10^{-5}$).

e,f Representative traces **(e)** and group data **(f)** showing that flufenamic acid (FLU), a NSCC blocker, nearly completely blocked the orexin-elicited inward current in the SLD neurons (ACSF: 22.8 ± 4.9 pA, Flu: 4.1 ± 0.5 pA; n = 6, two-sided pair-t test; $t_5 = 3.583$, P = 0.0158).

g The NSCC mainly contains Na^+ and K^+ conductance. We thus used the reversal potentials and the Goldman-Hodgkin-Katz equation to calculate the Na^+/K^+ permeability ratio of the orexin-elicited current on SLD neurons. Group data showing no differences in the 152.25 and 70 mM Na^+ ACSF (152.25 mM Na^+: 0.324 ± 0.040, n = 8; 70 mM Na^+: 0.311 ± 0.025, n = 7; two-sided Mann-Whitney rank-sum test; z =

0.231, P = 0.817). This suggests that the orexin-induced inward current may be solely mediated by the activation of NSCC.

Data represent mean ± SEM. *P < 0.05; **P < 0.01. Source data are provided as a Source Data file.

Supplementary Figure 8

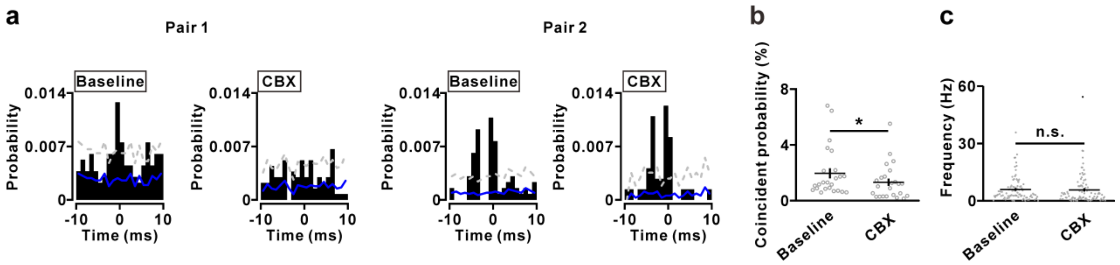

Supplementary Fig. 8 Microinjection of CBX into the SLD decreased the coincident probability in the SLD unit pairs

a Two example cross-correlograms from rat SLD unit pairs with significant coincidental spiking on the timescale of ±1 ms before microinjection of CBX (100 mM, 0.3 μl) into the SLD. CBX abolished coincidental spiking in pair 1, but did not affect coincidental spiking in pair 2.

b In consistent with experimental observations in **(a)**, group data showing that the coincident spiking probability in the SLD unit pairs was only partially decreased by CBX (baseline: 2.0 ± 0.4%, CBX: 1.3 ± 0.3%; n = 12 pairs with significant interactions in both baseline and CBX conditions; two-sided Wilcoxon signed-rank test; z = 2.386, P = 0.0170)

c Group data showing that CBX injection did not influence the frequency of the firing activities in the SLD units (baseline: 6.0 ± 0.7 Hz, CBX: 5.7 ± 0.8 Hz; n = 89 units, two-sided Wilcoxon signed-rank test; z = 1.395, P = 0.163).

Data represent mean ± SEM. *P < 0.05; Source data are provided as a Source Data file.

Supplementary Figure 9

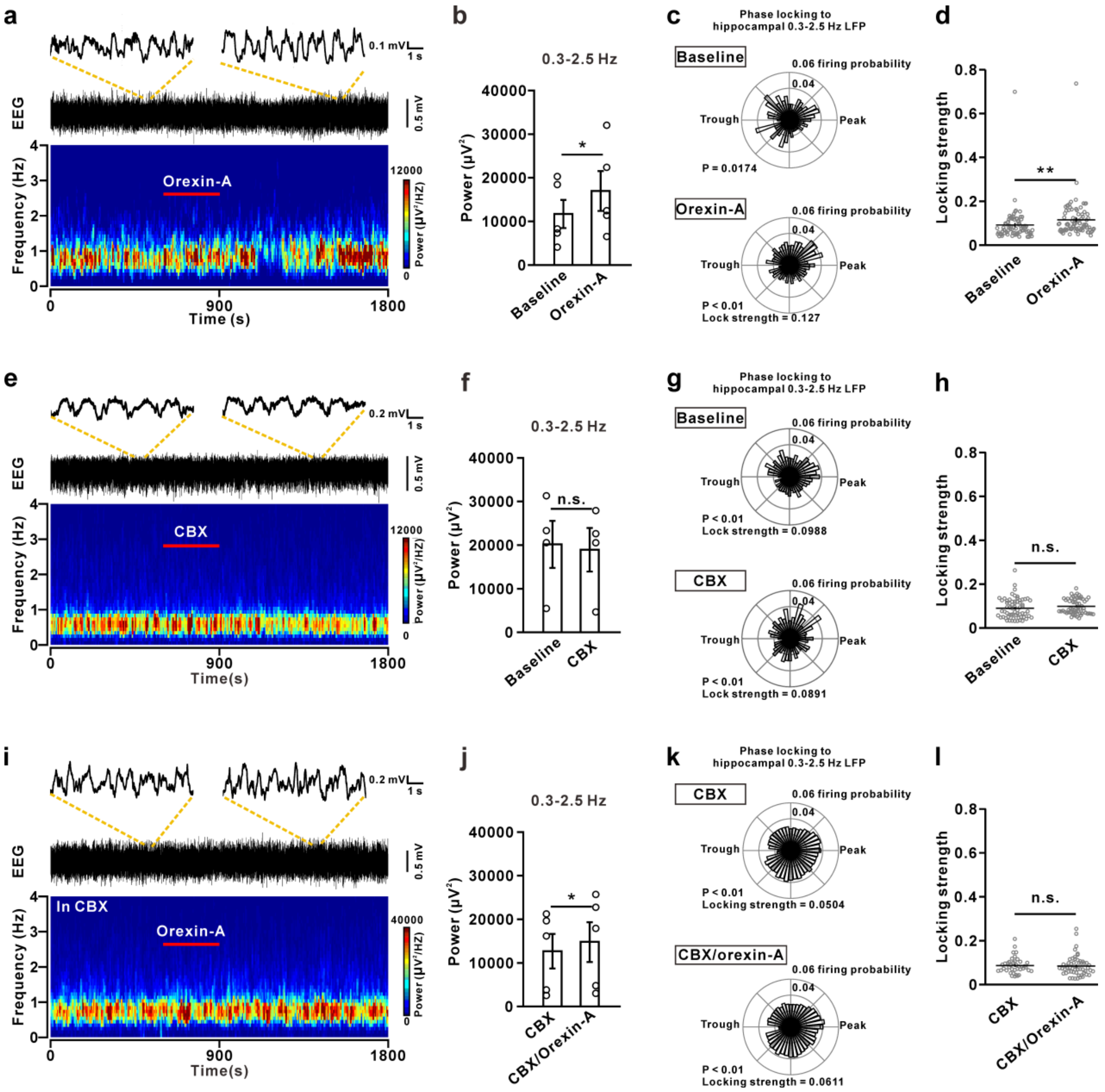

Supplementary Fig. 9 The orexin-elicited synchronized excitation contributes to the output of SLD neuronal network

a Power spectrogram and raw EEG traces of hippocampal recordings before and after microinjection of orexin-A (30 μM, 0.3 μl) into the SLD. During urethane anesthesia, the hippocampal activities were dominated by slow oscillations (0.3-2.5 Hz). Waveforms of this slow oscillations (in 10-second epoch) before and after

orexin-A microinjection were expanded at the top. An occasionally appeared disruption of hippocampal slow oscillations by a transient desynchronized/active state in anesthesia tends to increase after orexin-A microinjections.

b Group data showing the power of hippocampal slow oscillations (0.3-2.5 Hz) was increased after microinjection of orexin-A into SLD (baseline: 11688.4 ± 3224.7 μV^2, orexin-A: 16959.5 ± 4566.8 μV^2; n = 5 rats, two-sided pair-t test; t_4 = 3.196, P = 0.0330).

c Phase locking analysis between the SLD spiking activities and hippocampal slow oscillations (0.3 - 2.5 Hz) before and after orexin-A microinjections. Note that a significant phase preference emerged in the presence of orexin-A.

d Group data showing that the locking strength between SLD spiking activities and hippocampal slow oscillations was significantly increased after microinjection of orexin-A into the SLD (baseline: 0.092 ± 0.009, n = 76/97 pairs; orexin-A: 0.115 ± 0.009, n = 84/97 pairs; two-sided Mann-Whitney rank-sum test; z = 3.335, P = 8.533×10^{-4}).

e Power spectrogram and raw EEG traces of hippocampal recordings before and after microinjection of CBX (100 mM, 0.3 μl) into the SLD.

f Group data showing that CBX did not influence the power of hippocampal slow oscillation (0.3-2.5 Hz) (baseline: 20164.1 ± 5403.9 μV^2, CBX: 18934.5 ± 5001.5 μV^2; n = 4 rats, two-sided pair-t test; t_3 = 1.000, P = 0.391).

g Phase locking analysis between the SLD spiking activities and hippocampal oscillations (0.3 - 2.5 Hz) before and after microinjection of CBX into the SLD.

h Group data showing that the locking strength between the SLD spiking activities and hippocampal slow oscillations remained unchanged after microinjection of CBX into the SLD (baseline: 0.091 ± 0.006, n = 65/89 pairs; CBX: 0.099 ± 0.004, n = 59/89 pairs; two-sided Mann-Whitney rank-sum test; z = 1.814, P = 0.0697).

i Power spectrogram and raw EEG traces of hippocampal recordings before and after microinjection of orexin-A (30 μM, 0.3 μl) into the SLD in the presence of CBX (100 mM).

j Group data showing that orexin-A still increased the power of hippocampal oscillations (0.3-2.5 Hz) in the presence of CBX (CBX: 12680.7 ± 3971.6 μV^2 orexin-A: 14834.0 ± 4641.0 μV^2; n = 5 rats, two-sided pair-t test; t_4 = 2.958, P = 0.0416). But note that the percentage increase of this oscillation power in CBX/orexin-A condition was largely reduced, compared to that induced by orexin-A microinjection alone (orexin-A: 48.9 ± 7.0%, CBX/orexin-A: 18.4 ± 2.5%; n = 5 rats for each group, Mann-Whitney rank-sum test; z = 2.402, P = 0.0163), suggesting the involvement of the orexin-elicited synchronized excitation in the SLD output.

k Phase locking analysis between the SLD spiking activities and hippocampal oscillations (0.3 - 2.5 Hz) before and after microinjection of orexin-A into the SLD in the presence of CBX.

l Group data showing that the orexin-A induced increase in the locking strength between SLD spiking activities and hippocampal slow oscillations was abolished in the presence of CBX (baseline: 0.086 ± 0.006, n = 40/72; orexin: 0.084 ± 0.006, n = 55/72; two-sided Mann-Whitney rank-sum test; z = 0.693, P = 0.488).

Data represent mean ± SEM. *P < 0.05; **P < 0.01. Source data are provided as a Source Data file.

Supplementary Figure 10

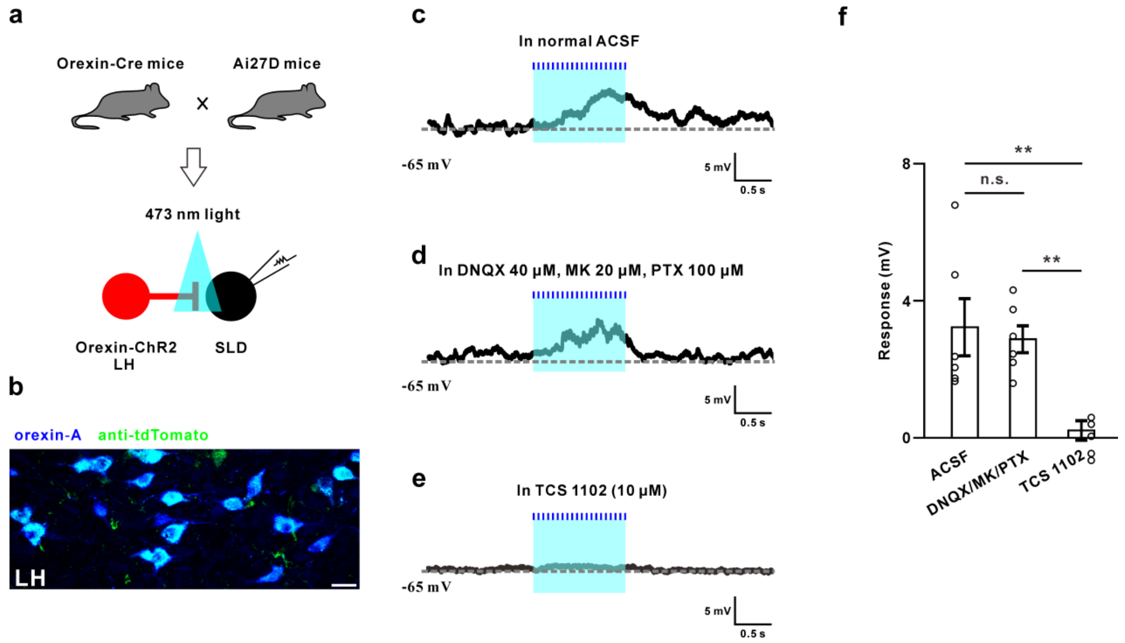

Supplementary Fig. 10 Optogenetic activation of the SLD orexin terminals depolarizes SLD neurons, and this depolarization is mediated by the release of orexin

a Schematic drawing for optogenetic activation of the SLD orexin terminals in brain slices from the orexin-Cre;Ai27D offspring.

b The Ai27D mice express a ChR2(H134R)/tdTomato fusion protein in a Cre-dependent manner. ChR2-tdTomato (green) expression in the LH orexin-A^{+} (blue) neurons was found at the age (8-14 days) of the used orexin-Cre;Ai27D offspring (scale bar, 20 μm).

c Optical (473 nm) activation (20 Hz; pulse width: 5ms; duration: 1 s) of the SLD orexin terminals elicited a depolarization in the tested SLD neuron under normal

ACSF condition.

d Optical activation of the SLD orexin terminals still elicited a depolarization in the tested SLD neuron after adding DNQX 40 μM, MK 801 (MK) 20 μM, and picrotoxin (PTX) 100 μM into the ACSF.

e In the presence of TCS 1102 (10 μM), optical activation of the SLD orexin terminals failed to elicited a depolarization in the tested SLD neuron.

f The optical-elicited responses in the SLD neurons under normal ACSF, DNQX/MK/PTX, and TCS 1102 conditions , respectively (normal ACSF: 3.2 ± 0.9 mV, DNQX/MK/PTX: 2.9 ± 0.4 mV; TCS 1102: 0.2 ± 0.3 mV; n = 6 neurons for each group; one-way ANOVA, $F_{(2, 15)} = 8.216$, $P = 3.894 \times 10^{-3}$; post-hoc LSD comparison test; ACSF vs DNQX/MK/PTX, P = 0.672; ACSF vs TCS 1102, $P = 2.111 \times 10^{-3}$; DNQX/MK/PTX vs TCS 1102, $P = 5.115 \times 10^{-3}$).

Data represent mean ± SEM. **P < 0.01. Source data are provided as a Source Data file.

Supplementary Figure 11

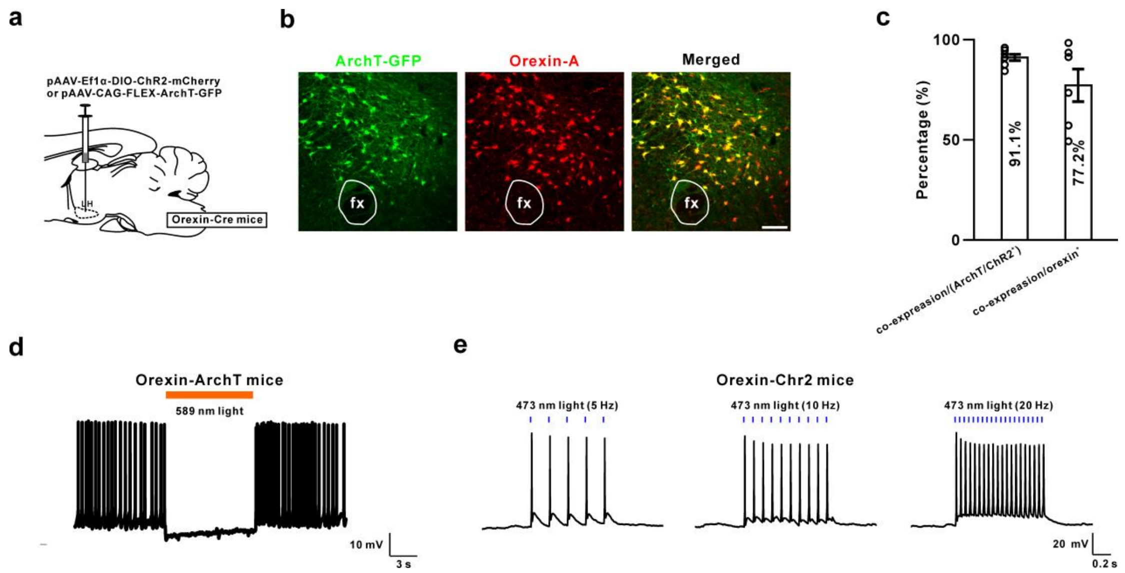

Supplementary Fig. 11 Morphological and electrophysiological validation of optogenetic manipulations

a AAV-Ef1α-DIO-ChR2-mCherry or AAV-CAG-FLEX-ArchT-GFP were injected into the LH of orexin-Cre mice, to selectively express ChR2-mCherry (orexin$^{\text{ChR2-mCherry}}$) or ArchT-GFP (orexin$^{\text{ArchT-GFP}}$) in the orexin-Cre mice, respectively.

b Representative images showing the expression of opsins in the LH orexin-A^{+} (blue) neurons (ArchT-GFP in this mice, green). Scale bar, 100 μm. fx, fornix.

c Quantification of the percentage of Orexin-A^{+} and ArchT/ChR2^{+} neurons in the ArchT/ChR2^{+} neurons and Orexin-A^{+} neurons, respectively, in orexin-Cre mice after virus injections (n = 6 mice; 3 orexin$^{\text{ArchT-GFP}}$ and 3 orexin$^{\text{ChR2-mCherry}}$ mice).

d Light (589 nm) stimulation reliably abolished the firing activities in the orexin neurons of the orexin^{ArchT} mice (experiments were repeated in 6 orexin-ArchT^{+}

neurons).

e 5, 10 and 20 Hz light (473 nm) stimulation reliably evoked action potentials in the orexin neurons of the orexin^{ChR2} mice (experiments were repeated in 6 orexin-ChR2^{+} neurons).

Data represent mean ± SEM. Source data are provided as a Source Data file.

Supplementary Figure 12

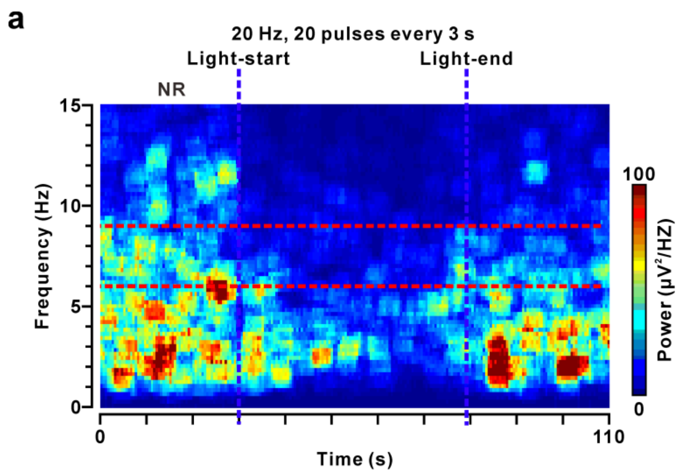

Supplementary Fig. 12 EEG power spectrogram before and after optogenetic activation of the SLD orexin terminals during NREM sleep

a EEG power spectrogram form a tested orexin^{ChR2} mouse shows that high amplitude theta oscillations characterizing REM sleep were not induced after optogenetic activation (20 Hz, 20 pulses every 3 s) of the SLD orexin terminals during NREM sleep, suggesting that light delivery failed to induce direct transitions to REM sleep in this condition.

Supplementary Figure 13

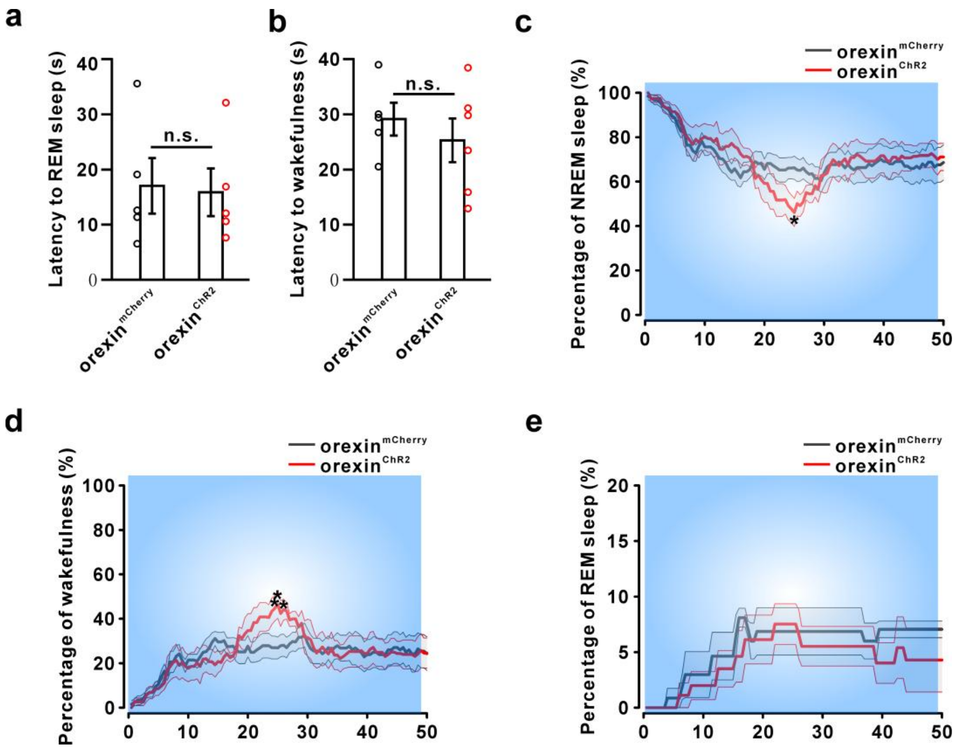

Supplementary Fig. 13 Percentage distributions of sleep/wakefulness occurrences after optogenetic activation (20 Hz, 20 pulses every 3 s) of the SLD orexin terminals during NREM sleep

a The mean latency to REM sleep was not changed after light delivery in NREM sleep (orexin^{mCherry}: 16.9 ± 5.0 s, n = 5 mice; orexin^{ChR2}: 15.8 ± 4.3 s, n = 5 mice; two-sided unpaired t-test, t_8 = 0.176, P = 0.864).

b The mean latency to wakefulness was not changed after light delivery in NREM sleep (orexin^{mCherry}: 29.0 ± 3.0 s, n = 5 mice; orexin^{ChR2}: 25.1 ± 3.9 s, n = 6 mice; two-sided unpaired t-test, t_9 = 0.749, P = 0.473).

c Percentage distribution of NREM sleep occurrences in 0.5 s bins showed that the

NREM sleep occurrences tended to decrease between ~20 s - ~30 s after the light delivery (orexin^{mCherry}: n = 5 mice; orexin^{ChR2}: n = 6 mice; one-way repeated measure ANOVA reported no significant main effect of light or time point among this time range, but a significant interaction between them; light: $F_{(1, 9)}$ = 2.105, P = 0.181; time point: $F_{(20, 180)}$ = 1.099, P = 0.353; interaction: $F_{(20, 180)}$ = 2.050, P = 7.205 $\times 10^{-3}$; post hoc LSD comparison test revealed a significant change at the 25 s, P = 0.0404). The period of light delivery was indicated by the blue background.

d Percentage distribution of wakefulness occurrences in 0.5 s bins showed that the wakefulness occurrences tended to increase between ~20 s - ~30 s after the light delivery (orexin^{mCherry}: n = 5 mice; orexin^{ChR2}: n = 6 mice; one-way repeated measure ANOVA reported no significant main effect of light or time point among this time range, but a significant interaction between them; light: $F_{(1, 9)}$ = 2.646, P = 0.138; time point: $F_{(20, 180)}$ = 0.908, P = 0.578; interaction: $F_{(20, 180)}$ = 1.670, P = 0.0419; post hoc LSD comparison test revealed a significant change at the 24.5 s, P = 0.0493; 25 s, P = 0.0402; 26 s, P = 0.0432).

e Percentage distribution of REM sleep occurrences in 0.5 s bins showing that the REM sleep occurrences were not changed after the light delivery (one-way repeated measure ANOVA reported no significant main effect of light or time point among this time range, and no significant interaction between them; light: $F_{(1, 9)}$ = 0.023, P = 0.882; time point: $F_{(20, 180)}$ = 0.820, P = 0.687; interaction: $F_{(20, 180)}$ = 0.820, P = 0.687). Data represent mean ± SEM. *P < 0.05. Source data are provided as a Source Data file.

Supplementary Figure 14

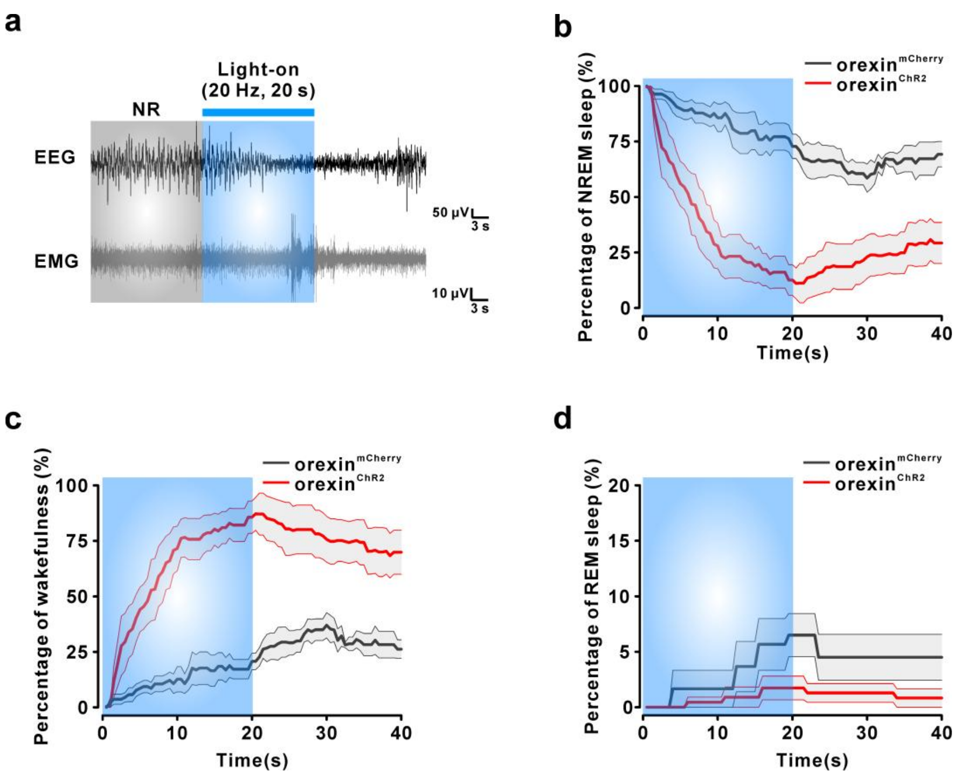

Supplementary Fig. 14 Percentage distribution of sleep/wakefulness occurrences after optogenetic activation (20 Hz for 20 s) of the SLD orexin terminals during NREM sleep

a An example trial from a tested orexin^{ChR2} mouse showing the changes of EMG and EEG recordings induced by optical activation (20 Hz for 20 seconds) of SLD orexin terminals during NREM sleep. The period of light delivery was indicated by the blue background.

b Percentage distribution of NREM sleep occurrences in 0.5 s bins showed that the NREM sleep occurrences decreased after the light delivery (n = 5 orexin^{mCherry} mice and 5 orexin^{ChR2} mice).

c Percentage distribution showing that wakefulness occurrences was high during the optical stimulation, suggesting that the brain state activation was intensive in this condition (n = 5 orexin^{mCherry} mice and 5 orexin^{ChR2} mice). Consistently, several previous studies have reported that strong excitation of SLD through direct optogenetic activation or inhibition of vlPAG-SLD GABA signaling indeed facilitated brain state activation and may lead to wakefulness from NREM sleep[2,3].

d Percentage distribution of REM sleep occurrences in 0.5 s bins showing that the REM sleep occurrences decreased after the light delivery (n = 5 orexin^{mCherry} mice and 5 orexin^{ChR2} mice).

Data represent mean ± SEM. Source data are provided as a Source Data file.

Supplementary Figure 15

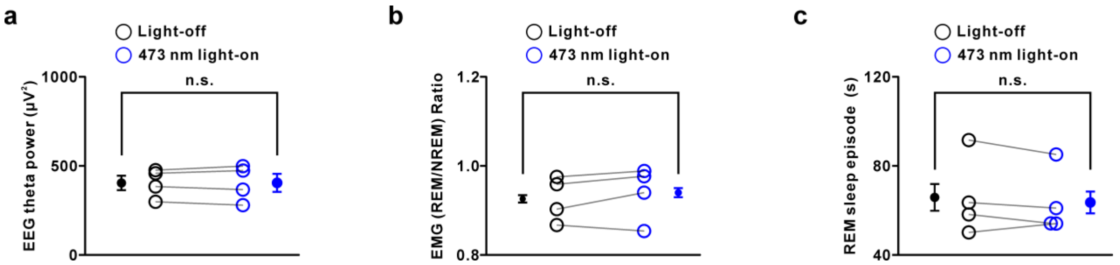

Supplementary Fig. 15 Optogenetic activation of the SLD orexin terminals during REM sleep had no effects in orexin^{mCherry} mice

a Optogenetic activation of the SLD orexin terminals during REM sleep did not influence the EEG theta power (light-off: 403.8 ± 40.5 μV^2, light-on: 404.5 ± 50.7 μV^2; n = 4 orexin^{mCherry} mice; two-sided pair-t test; t_3 = 0.063, P = 0.954).

b Optogenetic activation of the SLD orexin terminals during REM sleep did not influence the $EMG_{REM/NREM}$ ratio (light-off: 0.93 ± 0.03, light-on: 0.94 ± 0.03; n = 4 orexin^{mCherry} mice; two-sided pair-t test; t_3 = 1.317, P = 0.279).

c Optogenetic activation of the SLD orexin terminals during REM sleep did not influence the REM sleep episode duration (light-off: 65.9 ± 9.0 s, light-on: 63.6 ± 7.3 s; n = 4 orexin^{mCherry} mice; two-sided pair-t test; t_3 = 1.023, P = 0.382).

Data represent mean ± SEM. Source data are provided as a Source Data file.

Supplementary Figure 16

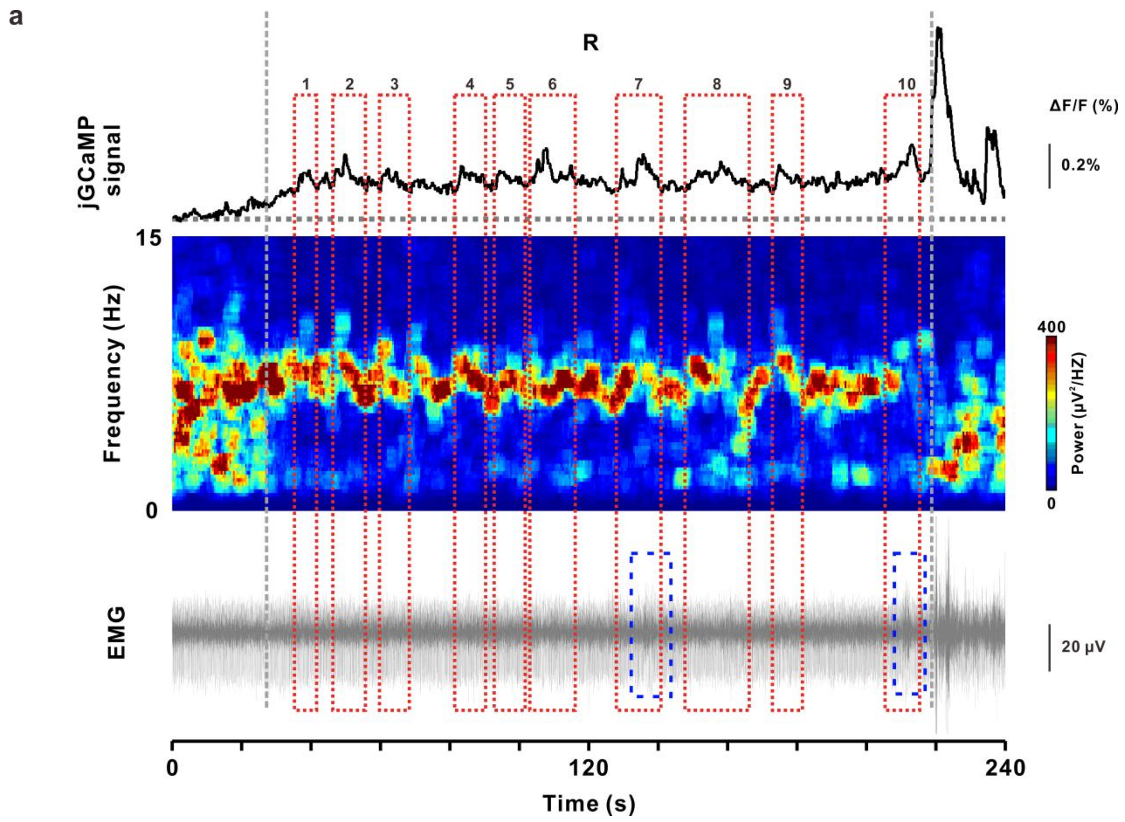

Supplementary Fig. 16 The jGCaMP transients of the SLD orexin terminals were observed during the entire REM sleep episodes

a Representative jGCaMP fluorescence traces of the SLD orexin terminals, spectrogram of EEG recordings, and raw EMG recorded simultaneously in a episode of REM sleep (R), the preceding NREM sleep (NR), and the following wakefulness (W). The red boxes labeled the time windows of ten detected jGCaMP transients (larger than 2 fold of the averaged signals), and the two blue boxes in the EMG recordings labeled the time windows of two set of muscle twitches. Note that the time windows in some jGCaMP transients (No. 7 and No. 10) seem to have a certain

degree of overlap with that of muscle twitches, while others did not, indicating that the jGCaMP signals of the SLD orexin terminals elevated during the time course of the entire REM sleep episodes, including both tonic and phasic periods.

Supplementary Figure 17

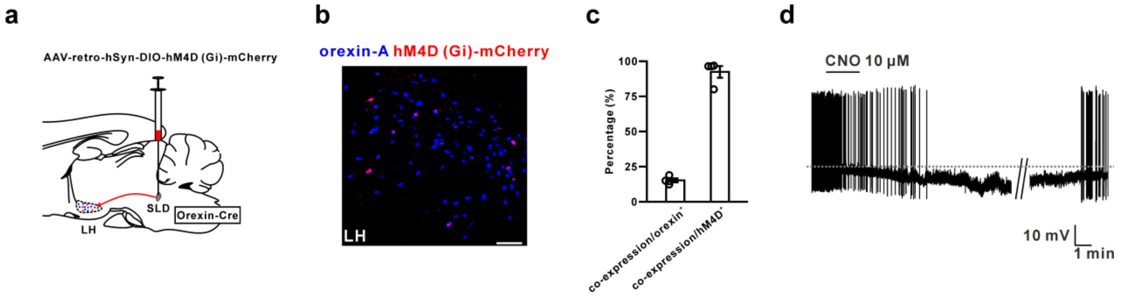

Supplementary Fig. 17 Morphological and electrophysiological validation of chemogenetic manipulations

a In chemogenetics, AAV-retro-hSyn-DIO-hM4D (Gi)-mCherry was bilaterally injected into the SLD of the orexin-Cre mice, to selectively label the orexin-SLD pathway.

b An example coronal image showing that virus injection in the SLD resulted in the expression of hM4D (Gi)-mCherry (red) in the SLD-projecting orexin neurons in the LH (orexin-A, blue). Scale bar, 100 μm.

c Quantification of the percentage of orexin-A^+/hM4D^+ neurons in all orexin-A^+ neurons (SLD-projecting orexin neurons, 15.2 ± 1.4%) and the percentage of orexin-A^+/hM4D^+ neurons in all hM4D^+ neurons (92.4 ± 4.1%) after the virus infections (n = 4 orexin$^{SLD\text{-}hM4D}$ mice).

d Patch-clamp recordings were performed on the hM4D (Gi)-mCherry$^+$ LH orexin neurons after 2-month of the virus injections in the brain slices. A 2-min bath application of CNO (10 μM) totally abolished the firing activities in all 6 identified

hM4D (Gi)-mCherry^{+} LH orexin neurons.

Data represent mean ± SEM. Source data are provided as a Source Data file.

Supplementary Figure 18

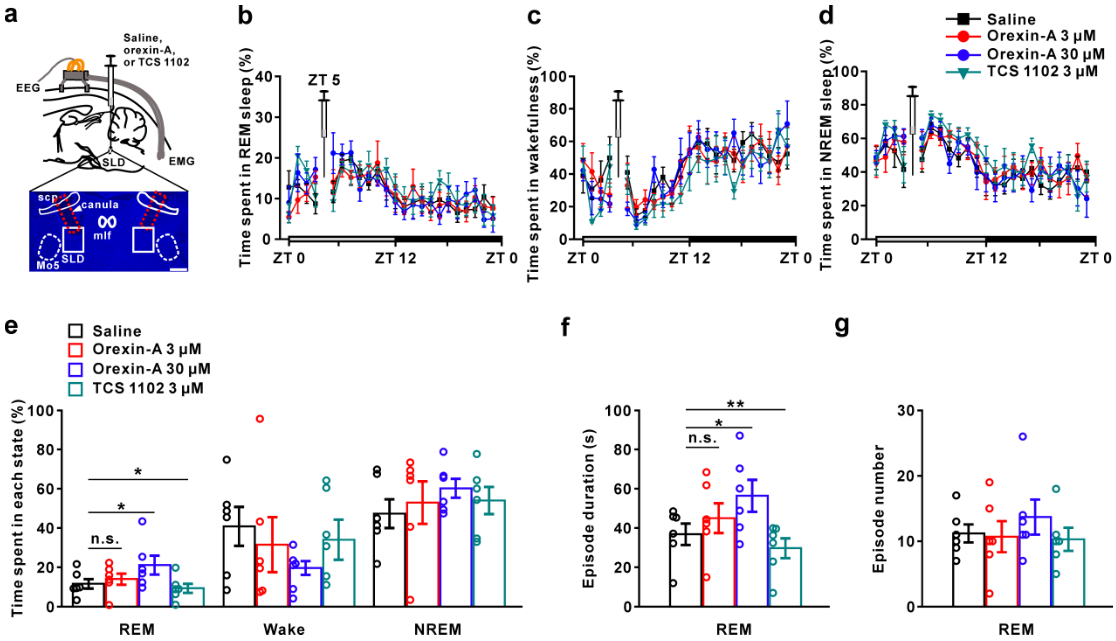

Supplementary Fig. 18 Microinjection of orexin-A and TCS 1102 into the SLD confirms a routinely-required role of SLD orexin signaling in the maintenance of REM sleep

a Schematic for the drug microinjections in SLD and EEG/EMG recordings in free-moving rats. The location of drug canulas in SLD were verified in all included rats and an example was shown below. Scale bar: 500 μm.

b-d Hourly percentage of time spent in REM sleep **(b)**, wakefulness **(c)**, and NREM sleep **(d)** before and after microinjections of 0.3 μl saline (black), orexin-A (3 μM, red), orexin-A (30 μM, blue), or TCS 1102 (3 μM, green) in the 24-hour sleep/wakefulness cycle (n = 6 rats). Needles indicated the time point (11:00-12:00 a.m.) of the drug microinjections. ZT 0: 8:00 a.m.. The sleep/wakefulness patterns

were not influenced by drug microinjections.

e Group data showing the percentage of time spent in each state during the following hour after drug injections (n = 6 rats). One-way repeated measure ANOVA reported significant changes of REM sleep amount ($F_{(3, 15)}$ = 7.684, P = 2.429 $\times 10^{-3}$), and no significant changes in wakefulness ($F_{(3, 15)}$ = 2.282, P = 0.121) and NREM sleep ($F_{(3, 15)}$ = 0.720, P = 0.555). Compared to the saline injection (11.7 ± 2.6%), 3 and 30 μM orexin-A injections dose-dependently increased REM sleep amount to 14.1 ± 3.0% and 21.2 ± 5.0%, respectively (post-hoc LSD comparison test; orexin-A 3 μM v.s. saline: P = 0.319; orexin-A 30 μM v.s. saline: P = 0.0468). Furthermore, TCS 1102 (3 μM) alone, a dual orexin receptor antagonist, decreased the REM sleep amount to 9.4 ± 2.5% (post-hoc LSD comparison test; TCS 1102 vs saline: P = 0.0304). These data were similar to that observed in chemogenetic experiments, confirming a routinely-required role of SLD orexin signaling in the maintenance of REM sleep amount.

f Group data showing the REM sleep episode duration during the following hour after drug injections (saline: 36.9 ± 5.6 s; orexin-A 3 μM: 45.1 ± 7.7 s; orexin-A 30 μM: 56.5 ± 8.3 s; TCS 1102 3 μM: 29.8 ± 5.2 s; n = 6 rats, one-way repeated measure ANOVA; $F_{(3, 15)}$ = 10.683, P = 5.194 $\times 10^{-4}$; post-hoc LSD comparison test; saline vs orexin-A 3 μM: P = 0.175; saline vs orexin-A 30 μM: P = 0.0262; saline vs TCS 1102 3 μM: P = 3.892 $\times 10^{-4}$).

g Group data showing the REM sleep episode number during the following hour after drug injections (saline: 11.2 ± 1.4; orexin-A 3 μM: 10.7 ± 2.4; orexin-A 30 μM: 13.7 ±

2.7; TCS 1102 3 μM: 10.3 ± 1.8; n = 6 rats, one-way repeated measure ANOVA; $F_{(3, 15)} = 2.173$, $P = 0.134$).

Data represent mean ± SEM. *P < 0.05; **P < 0.01. Source data are provided as a Source Data file.

Supplementary Figure 19

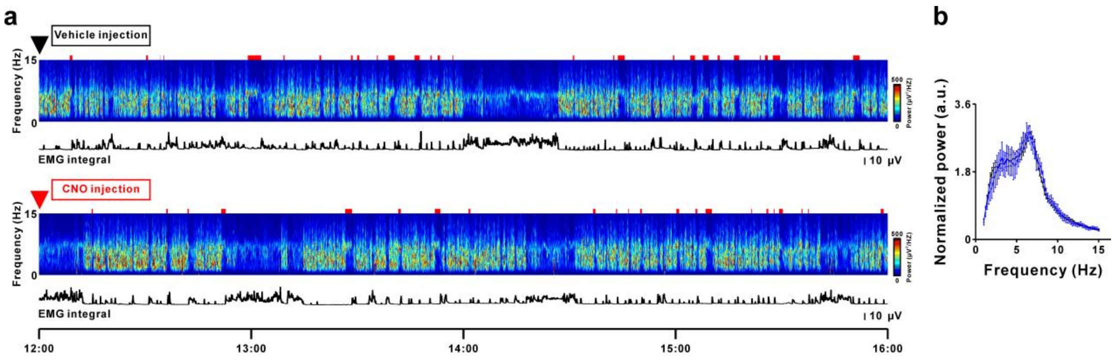

Supplementary Fig. 19 EEG activities after CNO or vehicle injections in orexin-Cre$^{SLD-hM4D}$ mice

a Color-indicated (red) REM sleep states, EEG power spectrogram, and EMG integral trace in an orexin-Cre$^{SLD-hM4D}$ mouse after vehicle and CNO injections.

b Power spectrum analysis of REM sleep episodes from **(a)** after vehicle (black) and CNO (blue) injections indicates SLD orexin signaling does not affect the dominant theta peak frequency during REM sleep.

Data represent mean ± SEM.

Supplementary Figure 20

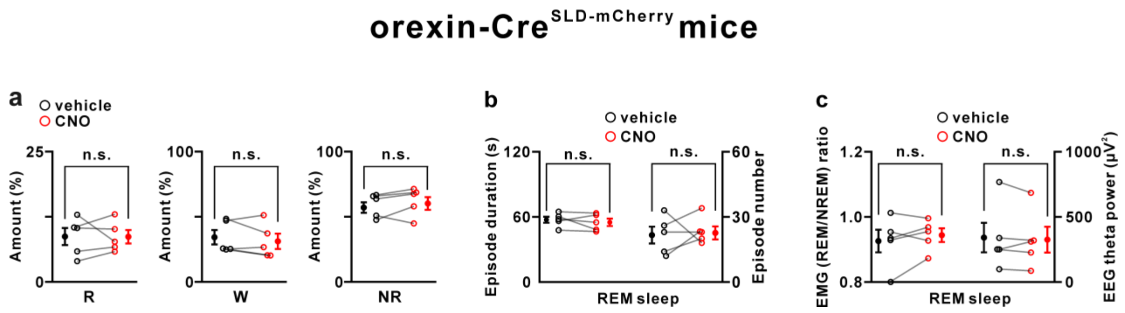

Supplementary Fig. 20 CNO injections did not influence REM sleep state in orexin-Cre$^{SLD-mCherry}$ mice

a CNO injections did not influence the amounts of REM sleep (R, vehicle: 8.7 ± 1.6%, CNO: 8.7 ± 1.3%; two-sided pair-t test; t_4 = 0.029, P = 0.979), wakefulness (W, vehicle: 34.3 ± 5.5%, CNO: 31.2 ± 5.9%; two-sided pair-t test; t_4 = 1.151, P = 0.314), and NREM sleep (NR, vehicle: 57.0 ± 4.0%, CNO: 60.2 ± 4.9%; two-sided pair-t test; t_4 = 1.193, P = 0.299) in orexin-Cre$^{SLD-mCherry}$ mice, compared to the vehicle injections (n = 5 mice).

b CNO injections did not influence the episode duration (left, vehicle: 57.4 ± 2.8 s, CNO: 54.9 ± 3.4 s; two-sided pair-t test; t_4 = 0.856, P = 0.440) and number (right, vehicle: 21.6 ± 3.9, CNO: 22.6 ± 3.0; two-sided pair-t test; t_4 = 0.237, P = 0.824) of REM sleep in orexin-Cre$^{SLD-mCherry}$ mice, compared to the vehicle injections (n = 5 mice).

c CNO injections did not influence the $EMG_{REM/NREM}$ ratio (left, vehicle: 0.93 ± 0.03, CNO: 0.94 ± 0.02; two-sided pair-t test; t_4 = 0.995, P = 0.376) and EEG theta power

(right, vehicle: 340.9 ± 113.3 μV^2, CNO: 324.9 ± 99.2 μV^2; two-sided pair-t test; t_4 = 0.707, P = 0.518) in orexin-Cre$^{SLD\text{-}mCherry}$ mice, compared to the vehicle injections (n = 5 mice).

Data represent mean ± SEM. Source data are provided as a Source Data file.

Supplementary References

1. Brown, R.E., McKenna, J.T., Winston, S., Basheer, R., Yanagawa, Y., Thakkar, M.M., and McCarley, R.W. (2008). Characterization of GABAergic neurons in rapid-eye-movement sleep controlling regions of the brainstem reticular formation in GAD67-green fluorescent protein knock-in mice. Eur. J. Neurosci. 27, 352-363.

2. Torontali, Z.A., Fraigne, J.J., Sanghera, P., Horner, R., and Peever, J. (2019). The sublaterodorsal tegmental nucleus functions to couple brain state and motor activity during REM sleep and wakefulness. 29, 3803-3813.

3. Weber, F., Hoang Do, J.P., Chung, S., Beier, K.T., Bikov, M., Saffari Doost, M., and Dan, Y. (2018). Regulation of REM and Non-REM Sleep by Periaqueductal GABAergic Neurons. Nat. Commun. 9, 354.
